# Identification of the promising *Ziziphus spina‐christi* (L.) Willd. genotypes using pomological and chemical proprieties

**DOI:** 10.1002/fsn3.2535

**Published:** 2021-08-16

**Authors:** Golnar Zandiehvakili, Ali Khadivi

**Affiliations:** ^1^ Department of Horticultural Sciences Faculty of Agriculture and Natural Resources Arak University Arak Iran

**Keywords:** antioxidant activity, total flavonoid content, total phenolic content, *Ziziphus*

## Abstract

*Ziziphus spina*‐*christi* (L.) Willd. is a multi‐purpose plant and is very popular found in local markets because of its high nutritional and medicinal values. The present work was carried out to study morphological and chemical properties of native accessions of this species. There were significant differences among the accessions investigated based on the morphological and chemical characters. Total phenolic content ranged from 4.84 to 49.58 mg/g fresh weight (FW). Total flavonoid content varied from 0.45 to 2.29 mg/g FW. Antioxidant activity measured with DPPH ranged from 0.32 to 16.99 mg/g FW, while it ranged from 6.64 to 84.15 µM FeSO_4_ FW with the FRAP method. The total phenol content showed significant and positive correlations with total flavonoid content (*r* = 0.33), antioxidant activity obtained with DPPH (*r* = 0.85), and antioxidant activity obtained with FRAP (*r* = 0.54). The ward dendrograms divided the accessions studied into two major clusters based on morphological and chemical characteristics. Based on the traits related to fruit quality such as fruit weight, fruit skin color, and fruit flavor, as well as in terms of chemical characteristics related to medicinal properties such as total flavonoids and antioxidant activity, 17 accessions were superior that could be used in breeding programs or cultivated directly. The present results can be used in defining conservation strategies, genetic improvement, and crop production.

## Graphical abstract

Based on the traits related to fruit quality, 17 accessions were superior that could be used in breeding programs or cultivated directly.

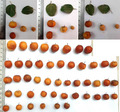

## 1 INTRODUCTION

The genus *Ziziphus* belongs to the Rhamnaceae family, which includes about 100 species of deciduous or evergreens with tree or shrub form. They are distributed throughout the tropical and subtropical regions of the world (Johnston, 1963), of which 12 species are cultivated (Hammer, 2001). One of the cultivated species of *Ziziphus* genus is *Z. spina‐christi* (L.) Willd. It is a shrub or spiny tree and has high resistance to drought and heat (National Academy of Sciences, 1980). The species is generally distributed at altitudes up to 600 m (Jongbloed, 2003; Saied et al., 2008) and grows well in desert areas with an annual rainfall of 50–300 mm (Maydell, 1986).

The *Z. spina‐christi* tolerates well in a variety of soils but performs best in light loamy soils (Vogt, 1995). In general, this species has a tree form, but it takes the form of a shrub or bush in dry and harsh conditions (Obeid & Mahmoud, 1971). Its roots can penetrate deep into the soil and are therefore highly tolerant of drought (Miehe, 1986). This plant is evergreen, but parts of its leaves fall off in very dry seasons (Maydell, 1986). Its height reaches between 5.00 and 10.00 m, and its trunk diameter is about 45.00 cm (El Amin, 1990; Saied et al., 2008).

Indigenous plants are part of the local agricultural and food source and are an important genetic source for maintaining biodiversity (Grivetti & Ogle, 2000). The potential of wild plant resources has not only been fully explored but has not been taken into account and has not been sufficiently involved in the supply of agricultural products (Saied et al., 2008; Schreckenberg et al., 2006). Local people have paid close attention to the importance of indigenous plants in terms of food supply, extra income, medicinal properties, and their use in preventing soil erosion (Gebauer et al., 2007). Due to the increasing population of the world and the need to feed them, the development of alternative crops is needed to improve the diet (El‐Siddig et al., 1999). Identifying and introducing crops that tolerate harsh conditions such as drought, heat, and salinity, is especially important (Saied et al., 2008).

Recently, attention to medicinal plants has been increased because they are pharmaceutical agents and are important for the development of drugs. According to the World Health Organization (WHO), traditional and herbal medicine is used by 80.00% of the world's population in developing countries for their primary healthcare (Alhakmani et al., 2014). The *Z. spina‐christi* is used to treat headaches, fractures, dandruff, chest pains, bruises, and blisters (Ghazanfar, 1994).

The *Z. spina‐christi*, known locally as Konar in Iran, is highly distributed in the southern regions of the country, such as Khuzestan, Bushehr, Fars, Kerman, Sistan, and Hormozgan provinces. This plant is multipurpose and is very popular in local markets due to its high nutritional and medicinal values. Also, native accessions of this species have high tolerance to heat, drought, and salinity (Miehe, 1986). Thus, they can play an important role in breeding programs related to the mentioned stresses. Due to its nutritional value and medicinal uses in traditional medicine, considerable attention has been paid to this plant. Therefore, it should be prevented from genetic erosion. Little is known about the morphological and chemical properties of this species in Iran. Thus, the current study was carried out to study morphological and chemical variation of some native accessions of this species selected from Shooshtar region in Khuzestan province from the south of Iran.

## 2 MATERIALS AND METHODS

### 2.1 Plant material

The present work was carried out to study morphological and chemical properties of 48 native accessions of *Z. spina*‐*christi* selected from Shooshtar region in Khuzestan province from the south of Iran. Shooshtar region is located at 31˚36'34"N latitude, 48˚35'22"E longitude, and 65 m height above sea level. To reduce the error and to prevent the collection of the clones belonging to a tree and ultimately increasing accuracy, a proper distance (200 m) between trees in each area was considered.

### 2.2 Morphological analysis

The phenotypic evaluations were carried out using 44 morphological and pomological characters. Fifty replicates for the leaf and fruit were used for measurements, and the mean values were used for analysis. The fruits were harvested at full ripening time based on taste and color and then transported to a laboratory. A digital caliper was used to measure dimensions‐related characters of leaf, thorn, fruit, and stone. Also, the weight of fruit and stone were measured using an electronic balance with 0.01 g precision. Furthermore, tree growth habit, tree vigor, tree height, branching, branch density, branch flexibility, trunk color, trunk type, trunk diameter, canopy symmetry, canopy density, tendency to suckering, thorn presence on current shoot, leaf density, leaf shape, leaf apex shape, leaf upper surface color, leaf lower surface color, leaf margin serration, leaf serration depth, fruit shape, fruit skin transparency, fruit skin color, fruit flesh color, fruit taste, fruit flesh texture, stone shape, stone surface, and stone terminal appendix were qualitatively estimated based on rating and coding.

### 2.3 Chemical analysis

#### 2.3.1 Total phenolic content

For extraction, samples (1.00 g) were homogenized with 10.00 ml of 80.00% methanol and the mixtures were centrifuged at 11200*g* G‐force for 10 min. Supernatants were collected and analyzed for total phenolic content and antioxidant activity assays. Total phenolic contents of fruit extracts were measured using the Folin‐Ciocalteu reagent method with spectrophotometry (Singleton & Rossi, 1965). Briefly, 400 μl of the extract was combined with 2.00 ml of ten‐fold–diluted Folin‐Ciocalteu reagent and 1.60 ml of sodium carbonate 7.50% and then placed at room temperature for 30 min. The absorbance was estimated at 756 nm. The concentration of total phenolic content was read in mg gallic equivalents per g fresh weight (FW) using a calibration curve prepared with gallic acid.

#### 2.3.2 Total flavonoid content

For determination of total flavonoid content, the method described by Grzegorczyk‐Karolak et al. (2015) was adopted so that the 2.00 ml of fruit extracts were mixed with 2.00 ml of 2.00% AlCl_3_ and the reaction mixture was allowed to stand for 15 min at room temperature. The absorbance was measured at 415 nm, and the findings were expressed as mg quercetin equivalents per g FW (mg QE/g FW) for total flavonoid content.

#### 2.3.3 Radical scavenging activity

The scavenging activity of the extracts prepared on 2,2‐diphenyl‐1‐picryl‐hydrazyl‐hydrate (DPPH) free radicals was determined. The 25 μl of the fruit extract was reacted with a 0.10 mM methanol solution of DPPH in a total volume of 3.00 ml, and the mixture was then placed in the dark at room temperature for 30 min. The absorbance was read at 517 nm. The DPPH scavenging activities were calculated based on the following formula: $\text{DPPH}\;\text{scavenging}\;\text{effect}\;\left(\%\right)=\left(A_{\text{control}}‐A_{\text{sample}}/A_{\text{control}}\right)\times 100$, where A_control_ and A_sample_ represent the control absorbance and the sample absorbance, respectively (Zhu et al., 2009). The DPPH scavenging activity of fruits was expressed as mg ascorbic acid equivalents per g FW using the established ascorbic acid calibration curve.

#### 2.3.4 Ferric reducing antioxidant power (FRAP)

The method developed by Benzie and Strain (1996) was used for the FRAP assay. The FRAP reagent comprised 300 mM acetate buffer, 10 mM TPTZ (2, 4, 6‐tripyridyl‐s‐triazine) in 40 mM HCl, and 20 mM ferric chloride (10:1:1, v/v/v). Three milliliters of FRAP reagent were added to 20 μl of fruit extract, and the reaction mixtures were placed in a 37℃ water bath for 10 min. The absorbance was read at 593 nm, and antioxidant activities were determined using the prepared FeSO_4_ standard curve.

### 2.4 Statistical analysis

Significant differences among the accessions in terms of the traits measured were determined using one‐way analysis of variance (ANOVA) with SAS software (SAS institute, 1990). The parameters, including minimum, maximum, mean, standard deviation (*SD*), and coefficient of variation (CV), were calculated. Pearson correlation coefficient was used to determine the correlation between traits with SPSS^®^ software version 16 (SPSS Inc. Norusis, 1998). The relationship between the accessions was analyzed using principal component analysis (PCA) with SPSS software. Hierarchical cluster analysis (HCA) was performed using Euclidean distance coefficient and Ward method with PAST software (Hammer et al., 2001). Distance coefficients were standardized using the Z scale. Also, the two‐dimensional plot was generated using the first and second principal components (PC1 and PC2) with PAST software.

## 3 RESULTS AND DISCUSSION

### 3.1 Morphological evaluations

There were significant differences among the accessions investigated based on the characters recorded (ANOVA, *p* < 0.01). The highest CVs belonged to stone terminal appendix (720.00%), tendency to suckering (149.33%), canopy symmetry (128.68%), and thorn number on annual shoot (115.88%). In contrast, stone width (9.96%), stone length (10.59%), fruit length (10.99%), and fruit width (12.29%) showed the lowest CVs. In total, 35 out of 44 characters measured had the CVs more than 20.00%, indicating high variability among the accessions (Table 1).

**TABLE 1:** Descriptive statistics for morphological traits utilized in the studied *Z. spina‐christi* accessions

No.	Character	Unit	Min.	Max.	Mean	*SD*	CV (%)
1	Tree growth habit	Code	1	5	2.08	1.16	55.96
2	Tree vigor	Code	1	5	3.62	1.38	38.07
3	Tree height	Code	3	5	3.67	0.95	25.97
4	Branching	Code	1	5	4.29	1.20	28.02
5	Branch density	Code	1	5	4.00	1.43	35.73
6	Branch flexibility	Code	1	5	3.21	1.25	39.07
7	Trunk color	Code	1	5	3.67	1.51	41.04
8	Trunk type	Code	1	3	2.21	0.99	44.71
9	Trunk diameter	Code	1	5	3.25	1.28	39.38
10	Canopy symmetry	Code	0	1	0.38	0.49	128.68
11	Canopy density	Code	1	5	3.88	1.30	33.48
12	Tendency to suckering	Code	0	5	0.75	1.12	149.33
13	Thorn presence on current shoot	Code	0	1	0.60	0.49	82.33
14	Thorn number on annual shoot	Number	0	5	1.60	1.85	115.88
15	Thorn length on annual shoot	mm	0.00	10.04	2.66	2.64	99.32
16	Leaf density	Code	1	5	4.17	1.08	25.85
17	Leaf shape	Code	1	5	3.42	1.01	29.44
18	Leaf apex shape	Code	1	3	1.46	0.85	58.15
19	Leaf upper surface color	Code	1	5	3.54	1.22	34.46
20	Leaf lower surface color	Code	1	3	1.38	0.79	57.17
21	Leaf margin serration	Code	0	1	0.71	0.46	64.65
22	Leaf serration depth	Code	0	1	0.71	0.46	64.65
23	Leaf length	mm	23.68	45.41	33.93	5.80	17.08
24	Leaf width	mm	15.39	33.10	23.72	4.18	17.63
25	Petiole length	mm	2.91	11.91	7.02	2.16	30.83
26	Petiole thickness	mm	0.56	1.07	0.76	0.11	14.61
27	Fruit length	mm	10.97	17.84	13.60	1.49	10.99
28	Fruit width	mm	11.29	18.60	14.51	1.78	12.29
29	Fruit fresh weight	g	0.88	3.63	1.87	0.69	36.63
30	Fruit stalk length	mm	2.45	6.82	4.53	0.94	20.84
31	Fruit stalk diameter	mm	0.45	0.87	0.64	0.10	15.16
32	Fruit flesh thickness	mm	1.85	5.30	3.33	0.79	23.72
33	Fruit shape	Code	1	5	3.71	1.52	40.84
34	Fruit skin transparency	Code	1	5	2.50	1.13	45.20
35	Fruit skin color	Code	1	11	7.83	2.67	34.07
36	Fruit flesh color	Code	1	7	3.58	2.58	71.93
37	Fruit taste	Code	1	7	5.54	2.05	37.04
38	Fruit flesh texture	Code	1	3	1.92	1.01	52.45
39	Stone length	mm	8.01	12.25	9.64	1.02	10.59
40	Stone width	mm	6.73	10.08	8.39	0.84	9.96
41	Stone weight	g	0.17	0.84	0.43	0.14	33.26
42	Stone shape	Code	1	5	2.00	1.09	54.60
43	Stone surface	Code	1	5	4.88	0.64	13.11
44	Stone terminal appendix	Code	0	1	0.02	0.14	720.00

Tree growth habit showed three types, including spreading (24 accessions), semi‐erect (22), and erect (2). Tree vigor was low in 6, intermediate in 21, and high in 21 accessions, while tree height was intermediate in 32 and high in 16 accessions. Branching and branch density were predominantly high in the majority of accessions (34 and 30, respectively). Three types of trunk color were observed, including light brown (8 accessions), brown (16), and gray (24). Bark of trunk contains many different compounds, including lignins, tannins, and suberins. These reflect and absorb different wavelengths of light, which explains the variations in color. The 19 accessions were single trunk, while 29 accessions were multi‐trunk. The density of canopy and leaves was predominately high (25 and 29, respectively) (Table 2).

**TABLE 2:** Frequency distribution for the measured qualitative morphological characters in the studied *Z. spina‐christi* accessions

Character	Frequency (no. of accessions)						
	0	1	3	5	7	9	11
Tree growth habit	‐	Spreading (24)	Semi‐erect (22)	Erect (2)	‐	‐	‐
Tree vigor	‐	Low (6)	Intermediate (21)	High (21)	‐	‐	‐
Tree height	‐	‐	Intermediate (32)	High (16)	‐	‐	‐
Branching	‐	Low (3)	Intermediate (11)	High (34)	‐	‐	‐
Branch density	‐	Low (6)	Intermediate (12)	High (30)	‐	‐	‐
Branch flexibility	‐	Low (7)	Intermediate (29)	High (12)	‐	‐	‐
Trunk color	‐	Light brown (8)	Brown (16)	Gray (24)	‐	‐	‐
Trunk type	‐	Single (19)	Multi (29)	‐	‐	‐	‐
Trunk diameter	‐	Low (7)	Intermediate (28)	High (13)	‐	‐	‐
Canopy symmetry	Absent (30)	Present (18)	‐	‐	‐	‐	‐
Canopy density	‐	Low (4)	Intermediate (19)	High (25)	‐	‐	‐
Tendency to suckering	No (26)	Low (16)	Intermediate (5)	High (1)	‐	‐	‐
Thorn presence on current shoot	No (19)	Yes (29)	‐	‐	‐	‐	‐
Leaf density	‐	Low (1)	Intermediate (18)	High (29)	‐	‐	‐
Leaf shape	‐	Ovate (2)	Lanceolate (34)	Elliptical (12)	‐	‐	‐
Leaf apex shape	‐	Rounded (37)	Acute (11)	‐	‐	‐	‐
Leaf upper surface color	‐	Light green (4)	Green (27)	Dark green (17)	‐	‐	‐
Leaf lower surface color	‐	Light green (39)	Green (9)	‐	‐	‐	‐
Leaf margin serration	None (14)	Serrate (34)	‐	‐	‐	‐	‐
Leaf serration depth	None (14)	Low (34)	‐	‐	‐	‐	‐
Fruit shape	‐	Flat (8)	Round (15)	Ovate (25)	‐	‐	‐
Fruit skin transparency	‐	Low (15)	Intermediate (30)	High (3)	‐	‐	‐
Fruit skin color	‐	Maroon yellow (3)	Maroon (1)	Dark maroon (9)	Yellow brown (1)	Light brown (28)	Dark brown (6)
Fruit flesh color	‐	Cream‐yellow (19)	Cream (11)	Cream‐brown (3)	Light orange (15)	‐	‐
Fruit taste	‐	Sour (2)	Sour‐sweet (14)	Slightly sweet (1)	Sweet (31)	‐	‐
Fruit flesh texture	‐	Soft (26)	Crisp (22)	‐	‐	‐	‐
Stone shape	‐	Round (25)	Ovate (22)	Elongate (1)	‐	‐	‐
Stone surface	‐	Smooth (1)	Relatively smooth (1)	Coarse (46)	‐	‐	‐
Stone terminal appendix	Absent (47)	Present (1)	‐	‐	‐	‐	‐

Leaf shape showed three forms, including ovate (2 accessions), lanceolate (34), and elliptical (12), while leaf apex shape was rounded (37) and acute (11). Leaf upper surface color was light green in 4, green in 27, and dark green in 17 accessions, while leaf lower surface color was light green in 39 and green in 9 accessions. Leaf margin serration was not observed in 14 accessions, while it was serrated in 34 accessions with low depth (Table 2). Leaf length ranged from 23.68 to 45.41 mm, and leaf width varied from 15.39 to 33.10 mm. Petiole length varied from 2.91 to 11.91 mm, and petiole thickness ranged from 0.56 to 1.07 mm (Table 1). Baghazadeh‐Daryaii et al. (2017) reported the range of 16.00–65.60 mm for leaf length, 11.90–51.50 mm for leaf width, and 1.45–19.30 mm for petiole length in some accessions of *Z. spina*‐*christi*. Also, Norouzi et al. (2017) reported the range of 18.90–37.00 mm for leaf length, 11.30–25.90 mm for leaf width, and 3.00–7.80 mm for petiole length in 33 individuals of *Z. spina*‐*christi*.

Fruit shape was flat in 8, round in 15, and ovate in 25 accessions. Fruit length varied from 10.97 to 17.84 mm, fruit width ranged from 11.29 to 18.60 mm, and the range of fruit flesh thickness was from 1.85–5.30 mm. Fruit fresh weight ranged from 0.88 to 3.63 g with an average of 1.87. Fruit flesh percentage ranged from 62.90 to 93.10%. The range of fruit stalk length and diameter was from 2.45–6.82 mm and 0.45–0.87 mm, respectively. Stone length varied from 8.01 to 12.25 mm, stone width ranged from 6.73 to 10.08 mm, and the range of stone weight was from 0.17–0.84 g (Table 1). Differences in fruit size and weight under the same environmental and geographical conditions are probably the result of genetic effects (Karadeniz, 2002). Baghazadeh‐Daryaii et al. (2017) reported the range of 10.68–27.45 mm for fruit length, 10.50–24.80 mm for fruit width, 2.20–11.40 mm for fruit flesh thickness, 0.68–7.70 g for fruit weight, 7.22–17.80 mm for stone length, 6.20–12.50 mm for stone width, and 0.22–3.40 g for stone weight in some accessions of *Z. spina*‐*christi*. Also, Norouzi et al. (2017) reported the range of 12.30–16.90 mm for fruit length, 12.90–19.20 mm for fruit width, 1.10–3.08 g for fruit weight, 8.30–10.90 mm for stone length, 7.70–8.80 mm for stone width, and 0.32–0.62 g for stone weight in 33 individuals of *Z. spina*‐*christi*.

**FIGURE 1:**
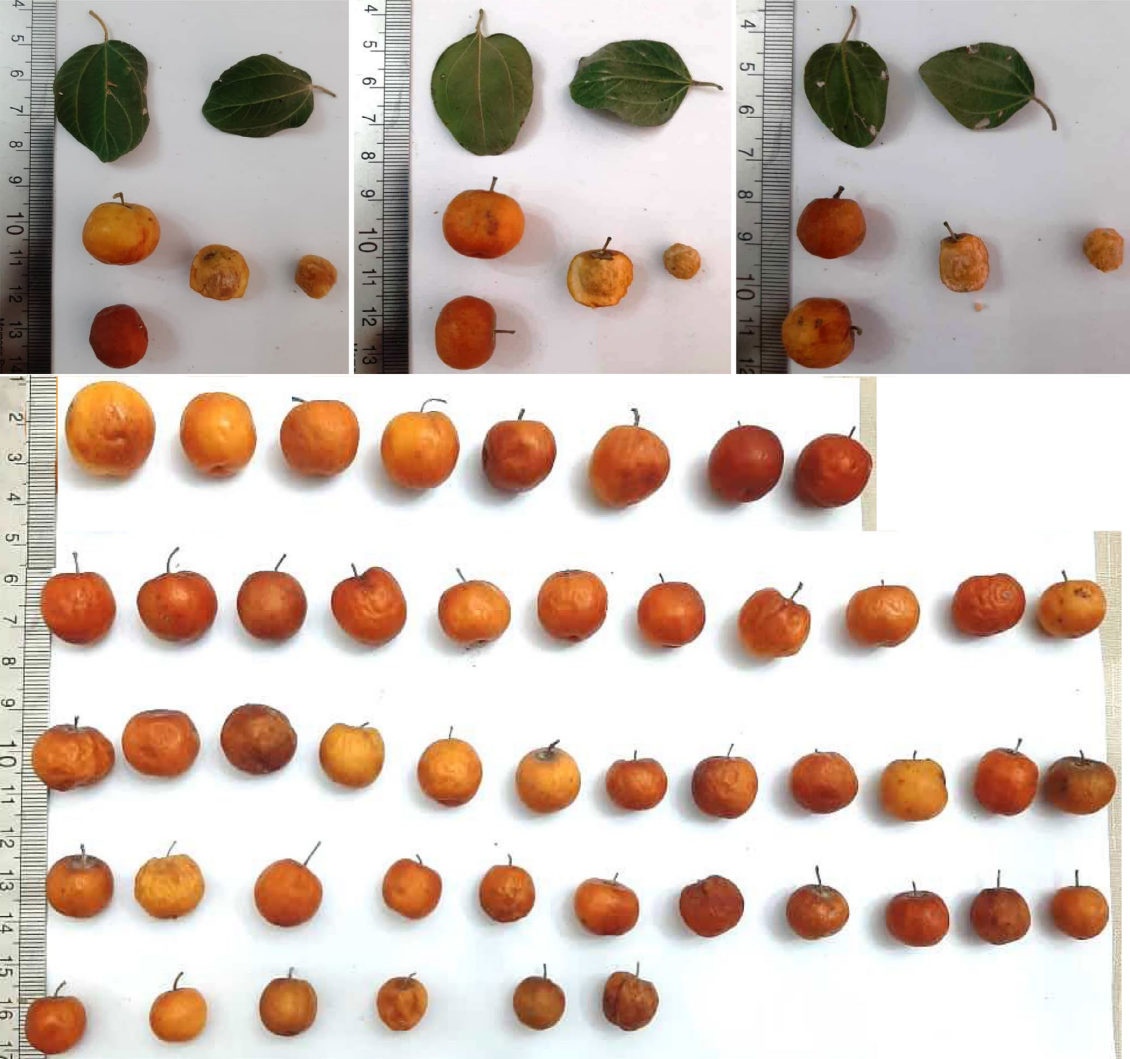
The pictures of leaves and fruits of *Z. spina‐christi* accessions studied

Fruit skin color showed high variations and included maroon yellow (3 accessions), maroon (1), dark maroon (9), yellow‐brown (1), light brown (28), and dark brown (6). Also, fruit flesh color was cream‐yellow (19 accessions), cream (11), cream‐brown (3), and light orange (15). Fruit taste was predominantly sweet (31 accessions), while other tastes such as sour (2), sour‐sweet (14), and slightly sweet (1) were observed. Fruit flesh texture was soft in 26 accessions, while it was crisp in 22 accessions. Stone shape was round in 25 and ovate in 22 accessions, while it was elongated only in one accession. Stone surface was predominantly coarse (46 accessions) (Table 2). The pictures of leaves and fruits of the studied accessions of *Z. spina*‐*christi* are shown in Figure 1.

There were significant correlations between some characters as revealed with Pearson coefficients (data not shown). Tree vigor was positively and significantly correlated with tree height (*r* = 0.52), branching (*r* = 0.51), branch density (*r* = 0.56), trunk diameter (*r* = 0.65), canopy density (*r* = 0.58), and leaf density (*r* = 0.46) and corresponded with the previous findings (Baghazadeh‐Daryaii et al., 2017; Norouzi et al., 2017). Leaf length showed significant and positive correlations with branching (*r* = 0.28), leaf shape (*r* = 0.32), leaf width (*r* = 0.80), petiole length (*r* = 0.76), and petiole thickness (*r* = 0.36) and agreed with the previous results (Baghazadeh‐Daryaii et al., 2017; Norouzi et al., 2017). Fruit fresh weight was positively and significantly correlated with leaf length (*r* = 0.65), leaf width (*r* = 0.54), petiole length (*r* = 0.41), and petiole thickness (*r* = 0.44). The leaves are responsible for absorbing sunlight for photosynthesis. Therefore, with increasing leaf size, leaf area is increased and then with increasing primary metabolites, suitable conditions are provided for the production of secondary metabolites (Khadivi‐Khub & Anjam, 2014).

Besides, fruit fresh weight showed positive and significant correlations with fruit length (*r* = 0.87), fruit width (*r* = 0.89), fruit stalk length (*r* = 0.52), fruit stalk diameter (*r* = 0.48), fruit flesh thickness (*r* = 0.86), stone length (*r* = 0.82), stone weight (*r* = 0.49), stone shape (*r* = 0.60), and stone terminal appendix (*r* = 0.64), in agreement with the previous results (Baghazadeh‐Daryaii et al., 2017; Norouzi et al., 2017). It has been reported that fruit weight and seed weight have high heritability coupled with high genetic advance in *Ziziphus* genus, and these traits are controlled by additive gene action, and phenotypic selection may be effective in improving them (Markovski and Velkoska‐Markovska, 2015).

The PCA reduces the number of correlated traits to a small number of components and interprets the relationship between variables (Khadivi, 2018). The PCA showed that the first 14 components accounted for 80.97% of the total variance (Table 3). The characters, including fruit length, fruit width, fruit fresh weight, fruit flesh thickness, stone length, stone width, and stone weight, were found to be correlated with PC1, accounting for 16.60% of the total variance, called fruit size‐related characters and corresponded with findings of Norouzi et al. (2017). The PC2 included seven traits, including tree vigor, tree height, branching, branch density, trunk diameter, canopy density, and leaf density, with explaining 11.49% of the total variance, called vegetative‐related traits. Three characters, including leaf length, leaf width, and petiole length, formed the PC3, accounting for 7.42% of total variance, called leaf‐related traits. In the previous studies, PCA has shown the importance of fruit‐related traits in describing the diversity between genotypes of *Z. spina*‐*christi* (Baghazadeh‐Daryaii et al., 2017; Norouzi et al., 2017).

**TABLE 3:** Eigenvalues of the principal component axes from the PCA of morphological characters in the studied *Z. spina‐christi* accessions

Character	PC1	PC2	PC3	PC4	PC5	PC6	PC7	PC8	PC9	PC10	PC11	PC12	PC13	PC14
Tree growth habit	−0.09	−0.20	−0.09	0.12	−0.13	−0.08	−0.01	0.05	−0.06	0.79**	0.17	−0.04	0.04	−0.02
Tree vigor	0.02	0.77**	0.24	−0.15	−0.02	−0.06	−0.03	0.04	−0.09	−0.33	0.12	−0.23	0.11	0.06
Tree height	0.19	0.53**	0.40	0.10	0.18	0.00	0.21	−0.24	0.29	0.01	0.15	−0.17	0.01	−0.10
Branching	−0.20	0.62**	0.14	−0.03	−0.02	−0.07	−0.03	−0.14	0.16	−0.30	0.12	0.33	−0.06	−0.13
Branch density	−0.11	0.86**	0.04	0.02	0.15	−0.06	−0.01	0.20	−0.20	0.09	−0.05	0.10	0.01	−0.05
Branch flexibility	0.17	0.04	0.11	0.16	0.01	0.21	−0.06	0.02	−0.06	0.27	0.00	−0.02	0.65**	−0.11
Trunk color	−0.08	0.00	−0.24	0.03	−0.02	0.09	0.25	0.74**	−0.03	−0.17	0.14	0.11	0.19	0.06
Trunk type	−0.04	−0.12	−0.04	0.08	0.01	0.86**	0.02	0.08	−0.13	0.02	−0.01	−0.04	0.02	0.01
Trunk diameter	0.22	0.62**	0.07	−0.24	−0.05	−0.18	0.05	−0.13	0.25	−0.33	−0.05	−0.16	0.00	0.03
Canopy symmetry	0.04	0.06	−0.13	−0.03	−0.04	0.14	−0.19	−0.05	−0.68**	0.36	0.00	−0.15	0.06	−0.03
Canopy density	0.08	0.71**	−0.03	−0.27	0.28	0.05	0.05	0.03	−0.18	−0.10	−0.24	0.09	0.10	0.02
Tendency to suckering	0.01	0.06	0.17	0.02	0.24	0.62**	−0.06	0.07	0.15	−0.07	−0.03	0.12	0.28	0.16
Thorn presence on current shoot	0.01	−0.08	−0.12	0.89**	−0.18	0.02	−0.02	−0.02	0.01	0.01	−0.05	0.00	0.07	0.12
Thorn number on annual shoot	−0.08	−0.18	−0.25	0.83**	0.16	−0.01	−0.12	−0.04	0.10	0.08	0.00	−0.04	0.02	0.01
Thorn length on annual shoot	−0.08	−0.11	−0.11	0.88**	−0.06	0.01	−0.07	0.07	0.05	0.07	−0.03	−0.06	0.06	0.03
Leaf density	−0.07	0.77**	−0.01	−0.08	0.25	0.07	−0.16	−0.05	0.04	0.23	−0.04	−0.08	−0.03	−0.08
Leaf shape	−0.06	0.00	0.02	−0.18	0.04	0.05	0.92**	0.06	0.03	−0.05	0.06	0.17	−0.07	−0.07
Leaf apex shape	−0.08	−0.05	0.05	−0.02	0.10	−0.10	0.91**	−0.05	−0.03	0.02	0.09	−0.11	−0.15	0.01
Leaf upper surface color	−0.17	−0.16	0.14	−0.10	−0.02	−0.22	−0.06	−0.27	0.37	0.32	0.02	0.02	0.13	0.52**
Leaf lower surface color	0.11	−0.08	−0.01	0.20	0.09	0.16	−0.04	0.00	−0.15	−0.10	0.03	−0.08	−0.10	0.78**
Leaf margin serration	0.13	0.25	0.14	−0.04	0.91**	0.02	0.09	−0.04	0.01	−0.07	−0.06	0.03	0.02	0.05
Leaf serration depth	0.13	0.25	0.14	−0.04	0.91**	0.02	0.09	−0.04	0.01	−0.07	−0.06	0.03	0.02	0.05
Leaf length	0.09	0.08	0.84**	−0.28	0.15	0.05	0.09	−0.07	0.01	−0.02	0.06	0.14	−0.09	0.11
Leaf width	0.20	0.08	0.78**	−0.09	0.20	0.03	−0.21	0.00	0.10	0.05	0.03	−0.26	−0.05	0.17
Petiole length	0.03	0.15	0.87**	−0.16	0.00	0.03	0.06	0.01	−0.10	−0.09	0.04	0.03	−0.05	−0.13
Petiole thickness	0.02	−0.04	0.13	−0.04	−0.06	−0.15	−0.29	0.73**	0.17	0.24	−0.13	0.03	−0.22	−0.13
Fruit length	0.92**	−0.01	0.14	−0.10	0.05	−0.03	−0.02	−0.14	−0.04	0.08	0.20	0.05	0.02	0.00
Fruit width	0.91**	−0.05	0.09	−0.11	0.22	−0.02	−0.09	−0.16	0.02	0.07	−0.05	0.07	−0.06	−0.02
Fruit fresh weight	0.92**	−0.05	0.09	−0.05	0.22	0.03	−0.06	−0.10	−0.08	0.03	0.00	0.12	−0.04	−0.01
Fruit stalk length	0.27	−0.01	0.49	−0.06	0.02	0.06	0.33	−0.29	0.06	−0.08	−0.16	0.35	0.10	−0.21
Fruit stalk diameter	0.42	0.30	0.10	0.02	−0.01	−0.09	−0.08	0.42	0.20	0.31	0.03	−0.25	−0.23	−0.16
Fruit flesh thickness	0.59**	0.03	0.20	−0.17	0.33	0.12	−0.12	−0.38	−0.14	0.02	0.05	0.30	−0.12	0.11
Fruit shape	−0.21	−0.17	0.15	0.03	−0.13	−0.33	0.12	0.13	0.23	−0.14	0.59**	−0.27	0.07	−0.09
Fruit skin transparency	0.06	0.10	0.18	0.33	0.18	−0.17	−0.33	−0.04	−0.09	−0.01	0.32	0.38	−0.12	−0.28
Fruit skin color	−0.17	−0.02	−0.14	0.15	−0.01	0.08	−0.16	0.12	0.78**	0.12	−0.15	−0.03	−0.16	−0.12
Fruit flesh color	0.04	0.13	0.07	0.00	−0.35	0.20	0.18	−0.01	0.28	0.17	0.40	0.33	−0.01	0.24
Fruit taste	0.12	0.19	0.23	0.14	−0.29	0.44	0.06	−0.20	−0.16	0.39	−0.22	0.25	−0.06	0.17
Fruit flesh texture	−0.22	0.05	−0.03	0.12	0.10	−0.82**	0.02	0.17	−0.02	0.05	−0.02	0.01	−0.03	0.02
Stone length	0.83**	0.12	0.11	0.00	−0.09	0.02	0.10	0.13	−0.17	−0.09	0.31	−0.01	0.06	0.06
Stone width	0.84**	−0.06	−0.04	0.10	−0.13	0.11	0.01	0.23	0.09	−0.09	−0.27	−0.10	0.07	−0.06
Stone weight	0.87**	0.00	−0.02	0.05	−0.06	0.19	−0.05	0.10	−0.05	−0.17	−0.18	0.08	0.18	0.10
Stone shape	0.09	−0.04	−0.01	−0.10	−0.04	0.05	0.07	−0.02	−0.21	0.15	0.82**	−0.04	−0.05	0.05
Stone surface	−0.01	0.04	−0.24	0.03	0.03	0.02	−0.15	−0.03	−0.11	−0.14	−0.03	−0.01	0.82**	0.01
Stone terminal appendix	0.36	−0.14	−0.07	−0.19	0.06	0.07	0.08	0.16	0.12	−0.06	−0.20	0.71**	0.01	−0.08
Total	7.30	5.06	3.26	2.82	2.57	2.26	2.11	2.08	1.72	1.60	1.40	1.26	1.17	1.02
% of variance	16.60	11.49	7.42	6.40	5.85	5.14	4.79	4.73	3.92	3.62	3.17	2.87	2.66	2.32
Cumulative %	16.60	28.09	35.51	41.91	47.76	52.90	57.69	62.42	66.33	69.95	73.13	75.99	78.65	80.97

Note

** Eigenvalues ≥0.52 are significant.

**FIGURE 2:**
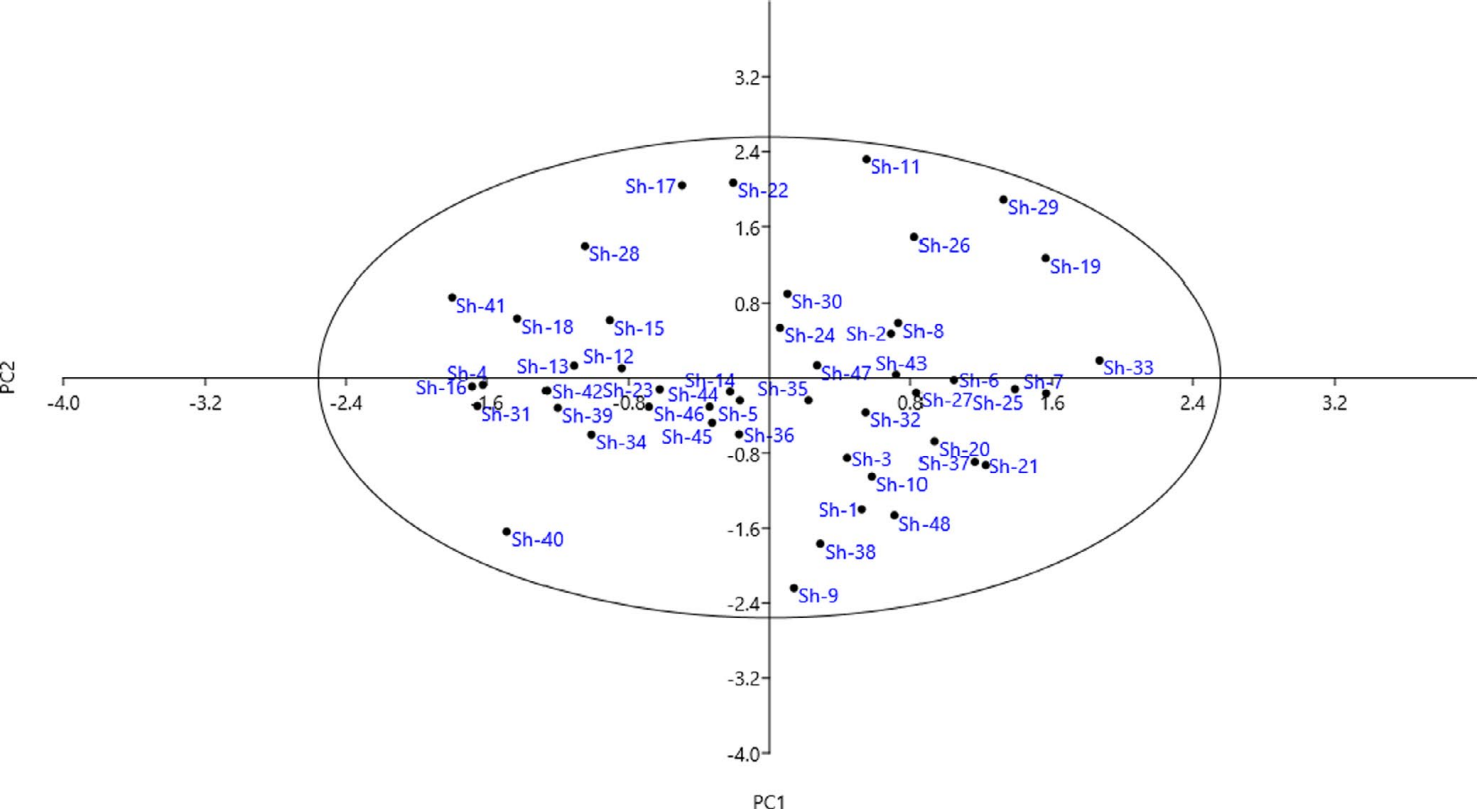
Scatter plot for the studied *Z. spina‐christi* accessions based on PC1/PC2 of morphological characters. The symbols represent the accessions of Shooshtar (Sh)

The scatter plot created using PC1/PC2 showed phenotypic variations among the accessions (Figure 2). Starting from negative to positive values of PC1, the accessions showed gradual increases in fruit length, fruit width, fruit fresh weight, fruit flesh thickness, stone length, stone width, and stone weight. Also, starting from negative to positive values of PC2, the characters, including tree vigor, tree height, branching, branch density, trunk diameter, canopy density, and leaf density, showed gradual increases among the accessions studied.

**FIGURE 3:**
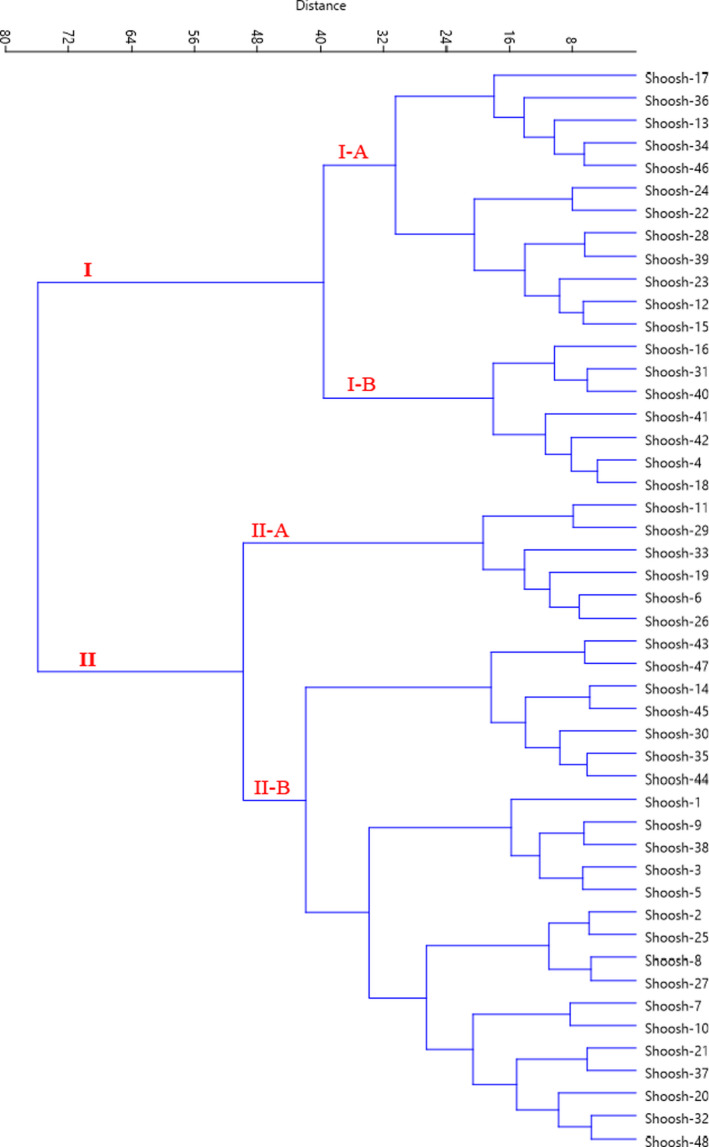
Ward cluster analysis of the studied *Z. spina‐christi* accessions based on morphological traits using Euclidean distances

Besides, HCA based on all the morphological data showed that the accessions studied were divided into two main clusters as revealed with the dendrogram created using Ward and Euclidean distance coefficients (Figure 3). The first cluster (I) was divided into two sub‐clusters. Sub‐cluster I‐A included 12 accessions, and sub‐cluster I‐B consisted of seven accessions. Also, sub‐cluster II‐A included six accessions, and sub‐cluster II‐B consisted of 23 accessions. There was high phenotypic diversity among the accessions. Self and cross‐incompatibility is a common and unique phenomenon in the genus *Ziziphus*, which leads to an increase in its genetic and phenotypic diversity apart from clonal and bud mutations (Weekley et al., 2002).

### 3.2 Chemical evaluations

The ANOVA revealed significant variations among the accessions studied in terms of the chemical properties measured (*p* < 0.01). Antioxidant activity measured with DPPH method showed the highest CV (108.32%), while total flavonoid content had the lowest CV (33.53%) (Table 4). Total phenolic content ranged from 4.84 to 49.58 mg/g FW. El Maaiden et al. (2018) reported that the value of total phenolic content in *Z. spina*‐*christi* was 39.74 mg GAE/g DW. Phenolic compounds play an important role in plants as primary antioxidants or free radical scavengers, and their antioxidant activity is due to their redox activity, which plays a key role in the uptake and sterilization of free radicals, quenching singlet and triple oxygen, and decomposition of peroxides (Abraham et al., 2006; Himesh et al., 2011). Phenolic compounds are a group of antioxidant agents that act as terminators of free radicals, and their bioactivity may be due to their ability to chelate metals, inhibit lipoxygenases, and free radical scavenging (Lin et al., 2005; Mallavadhani et al., 2006; Zheng & Wang, 2001). Phenolic compounds have also been reported to provide antimutagenic and anticarcinogenic properties in humans when approximately 1.00 g of them is consumed daily through a diet rich in vegetables and fruits (Tanaka et al., 1998).

**TABLE 4:** Descriptive statistics for the chemical traits utilized in the studied *Z. spina‐christi* accessions

No.	Character	Abbreviation	Unit	Min.	Max.	Mean	*SD*	CV (%)
1	Total phenolic content	Phe	mg/g FW	4.84	49.58	9.94	6.61	66.44
2	Total flavonoid content	Fla	mg/g FW	0.45	2.29	1.18	0.40	33.53
3	Antioxidant capacity (DPPH)	DPPH	mg/g FW	0.32	16.99	2.24	2.42	108.32
4	Antioxidant capacity (FRAP)	FRAP	*µ*MFeSo_4_ FW	6.64	84.15	35.08	16.22	46.23

Total flavonoid content varied from 0.45 to 2.29 mg/g FW. Baghazadeh‐Daryaii et al. (2017) reported the range of 1.39–3.66 mg/g for total flavonoid content in some accessions of *Z. spina*‐*christi*. El Maaiden et al. (2018) reported that the value of total flavonoid content in *Z. spina*‐*christi* was 0.02 mg QE/g DW. Brito et al. (2015) reported an average of 21.30 mg/g for flavonoid content in *Z. joazeiro*. Flavonoids are a group of phenolic compounds presented in fruits and vegetables that are characterized by a benzo‐y‐pyrone structure (Cheng et al., 2000). The antioxidant activity of flavonoids depends on the structure and substitution pattern of hydroxyl groups (Kaurinovic et al., 2011; Zheng & Wang, 2001). Flavonoids exited in herbs are important in human health because of their pharmacological activity as antioxidants and radical scavengers, which are particularly scavengers of most oxidizing molecules such as singlet oxygen and various free radicals (Schubert et al., 2007; Turkmen et al., 2006).

Antioxidant activity measured with DPPH ranged from 0.32 to 16.99 mg/g FW, while it ranged from 6.64 to 84.15 µM FeSO_4_ FW with the FRAP method (Table 4). Brito et al. (2015) reported low antioxidant activity with DPPH and FRAP in *Z. joazeiro*. Antioxidants are of interest to biologists and clinicians because they protect the human body from the damage caused by reactive free radicals in ischemic heart disease, cancer, Alzheimer, Parkinson, atherosclerosis, and even the aging process (Aruoma, 2003; Hemati et al., 2010).

There were significant correlations between chemical properties (Table 5). Total phenol content showed significant and positive correlations with total flavonoid content (*r* = 0.33), antioxidant activity obtained with DPPH (*r* = 0.85), and antioxidant activity obtained with FRAP (*r* = 0.54). The strong correlations have been reported between total phenolics and antioxidant activity in different fruits (Donno et al., 2015; Sachez‐Salcedo et al., 2015; Hosseini et al., 2018; Fereidoonfar et al., 2019; Krishna et al., 2020). Antioxidant activity obtained with DPPH was positively and significantly correlated with antioxidant activity obtained with FRAP (*r* = 0.77). High correlations have been shown between antioxidant activity obtained with DPPH and FRAP in different fruits (Krishna et al., 2020).

**TABLE 5:** Simple correlations among the chemical variables utilized in the studied *Z. spina‐christi* accessions

Character	Phe	Fla	DPPH	FRAP
Phe	1			
Fla	0.33*	1		
DPPH	0.85**	0.32*	1	
FRAP	0.54**	0.33*	0.77**	1

Note

^*^, ^**^ Correlation is significant at p ≤ 0.05 and 0.01 levels, respectively.

**FIGURE 4:**
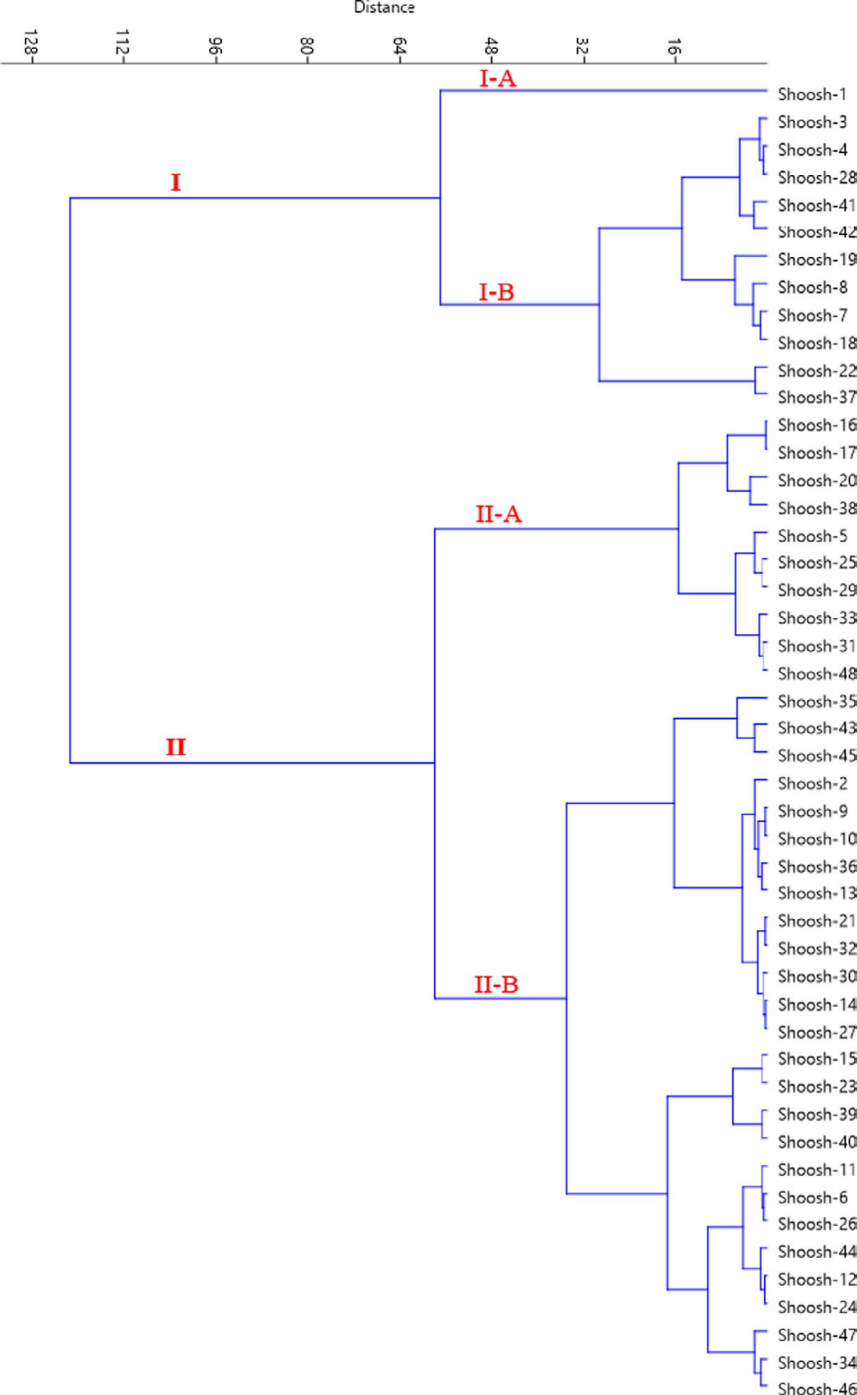
Ward cluster analysis of the studied *Z. spina‐christi* accessions based on chemical traits using Euclidean distances

The Ward dendrogram divided the accessions studied into two major clusters (Figure 4). The first cluster (I) was divided into two sub‐clusters. Sub‐cluster I‐A consisted of only Shooshtar‐1 accession, characterized by the highest values for total phenol content and antioxidant activity obtained with DPPH. Sub‐cluster I‐B consisted of 11 accessions, characterized by higher total phenol content and antioxidant activity obtained with DPPH. Also, the second cluster (II) was divided into two sub‐clusters. Sub‐cluster II‐A included 10 accessions, characterized by higher antioxidant activity obtained with FRAP. Sub‐cluster II‐B consisted of 26 accessions, characterized by higher total flavonoid content. The fruits of *Z. spina‐christi* are one of the most important horticultural crops because they are rich in nutritional values. The quantity of flavonoids that have protective effects on human health has been one of the primary sources of future industrial research in phytochemical studies of *Z. spina*‐*christi*.

## 4 CONCLUSION

The *Z. spina‐christi* is a multi‐purpose plant species that has high medicinal uses and can also be used as a pasture and landscape. Besides, due to the high nutritional value of its fruit, it can be considered as a horticultural crop. The present study showed that the accessions studied of this species have a high diversity in terms of morphological traits and chemical properties that can be widely used to introduce cultivars in breeding programs and the pharmaceutical industry. The obtained results can be used in selecting and introducing cultivars with the desired traits. Based on the traits related to fruit quality such as fruit weight, fruit skin color, and fruit flavor, as well as in terms of chemical characteristics related to medicinal properties such as total flavonoids and antioxidant activity, 17 accessions, including Shooshtar‐1, Shooshtar‐7, Shooshtar‐10, Shooshtar‐17, Shooshtar‐16, Shooshtar‐2, Shooshtar‐25, Shooshtar‐13, Shooshtar‐29, Shooshtar‐33, Shooshtar‐30, Shooshtar‐21, Shooshtar‐3, Shooshtar‐36, Shooshtar‐5, Shooshtar‐43, and Shooshtar‐20, were superior that could be used in breeding programs or cultivated directly. The present results can be used in defining conservation strategies, genetic improvement, and crop production.

## CONFLICT OF INTEREST

The authors declare no conflict of interest.

## AUTHOR CONTRIBUTIONS

**Golnar Zandievakili:** Investigation (equal). **Ali Khadivi:** Formal analysis (equal); Funding acquisition (equal); Investigation (equal); Supervision (equal); Validation (equal).

## RESEARCH INVOLVING HUMAN PARTICIPANTS AND/OR ANIMALS

None.

## INFORMED CONSENT

None.

## Data Availability

The data that support the findings of this study are available from the corresponding author upon reasonable request.

## References

[fsn32535-bib-0001] Abraham, R. , Carty, N. , DuFour, D. , & Pincus, M. (2006). In R. McPherson M. Pincus (Eds.), Clinical enzymology, in Henrys Clinical diagnosis and management by laboratory methods (21 ed.). Philadelphia: Sunders Elsevier.

[fsn32535-bib-0002] Alhakmani, F. , Khan, S. A. , & Ahmad, A. (2014). Determination of total phenol, in‐vitro antioxidant and anti‐inflammatory activity of seeds and fruits of *Zizyphus spina‐christi* grown in Oman. Asian Pac J Trop Biomed, 4(2), S656–S660. 10.12980/APJTB.4.2014APJTB-2014-0273

[fsn32535-bib-0003] Aruoma, O. I. (2003). Methodological considerations for characterizing potential antioxidant actions of bioactive components in plant foods. Mutation Research, 523–524, 9–20. 10.1016/s0027-5107(02)00317-2 12628499

[fsn32535-bib-0004] Baghazadeh‐Daryaii, L. , Sharifi‐Sirchi, G.‐R. , & Samsampoor, D. (2017). Morphological, phytochemical and genetic diversity of *Ziziphus spina–christi* (L.) Des. in South and Southeastern of Iran. Journal of Applied Research on Medicinal and Aromatic Plants, 7, 99–107. 10.1016/j.jarmap.2017.06.006

[fsn32535-bib-0005] Benzie, I. F. F. , & Strain, J. J. (1996). The ferric reducing ability of plasma (FRAP) as a measure of ‘antioxidant power’: The FRAP assay. Analytical Biochemistry, 239, 70–76. 8660627 10.1006/abio.1996.0292

[fsn32535-bib-0006] Brito, S. M. O. , Coutinho, H. D. M. , Talvani, A. , Coronel, C. , Barbosa, A. G. R. , Vega, C. , Figueredo, F. G. , Tintino, S. R. , Lima, L. F. , Boligon, A. A. , Athayde, M. L. , & Menezes, I. R. A. (2015). Analysis of bioactivities and chemical composition of *Ziziphus joazeiro* Mart. using HPLC–DAD. Food Chemistry, 186, 185–191. 10.1016/j.foodchem.2014.10.031 25976809

[fsn32535-bib-0007] Cheng , G. , Bai , Y. , Zhao , Y. , Tao , J. , Liu , Y. , Tu, G. , Ma , L. , Liao , N. , & Xu, X. (2000). Flavonoids from *Ziziphus jujube* Mill var. *spinosa* . Tetrahedron, 56, 8915–8920.

[fsn32535-bib-0008] Donno, D. , Cerutti, A. K. , Prgomet, I. , Mellano, M. G. , & Beccaro, G. L. (2015). Foodomics for mulberry fruit (*Morus* spp.): Analytical fingerprint as antioxidants’ and health properties’ determination tool. Food Research International, 69, 179–188.

[fsn32535-bib-0009] El Amin, H. M. (1990). Trees and shrubs of the Sudan. Ithaca Press Exeter.

[fsn32535-bib-0010] El Maaiden , E. , El Kharrassi , Y. , Moustaid, K. , Essamadi , A. K. , & Nasser , B. (2018). Comparative study of phytochemical profile between *Ziziphus spina christi* and *Ziziphus lotus* from Morocco. Journal of Food Measurement and Characterization., 13, 121–130. 10.1007/s11694-018-9925-y

[fsn32535-bib-0011] El‐Siddig, K. , Ebert, G. , & Ludders, P. (1999). Tamarind (*Tamarindus indica* L.): A review on a multipurpose tree with promising future in the Sudan. Angewandte Botanik, 73, 202–205.

[fsn32535-bib-0012] Fereidoonfar, H. , Salehi‐Arjmand, H. , Khadivi, A. , Akramian, M. , & Safdaria, L. (2019). Chemical variation and antioxidant capacity of sumac (*Rhus coriaria* L.). Industrial Crops and Products, 139, 111518. 10.1016/j.indcrop.2019.111518

[fsn32535-bib-0013] Gebauer, J. , El‐Siddig, K. , El‐Tahir, B. A. , Salih, A. A. , Ebert, G. , & Hammer, K. (2007). Exploiting the potential of indigenous fruit trees: *Grewia tenax* in Sudan. Genetic Resources and Crop Evolution, 54, 1701–1708.

[fsn32535-bib-0014] Ghazanfar, S. A. (1994). Handbook of arabian medicinal plants (pp. 182). CRC Press.

[fsn32535-bib-0015] Grivetti, L. E. , & Ogle, B. M. (2000). Value of traditional food in meeting macro‐ and micronutrients needs: The wild plant connection. Nutrition Research Reviews, 13, 31–46. 19087432 10.1079/095442200108728990

[fsn32535-bib-0016] Grzegorczyk‐Karolak, I. , Kuzma, L. , & Wysokinska, H. (2015). Study on the chemical composition and antioxidant activity of extracts from shoot culture and regenerated plants of *Scutellaria altissima* L. Acta Physiologiae Plantarum, 37, 1736.

[fsn32535-bib-0017] Hammer, K. (2001). Rhamnaceae. In P. Hanelt , & IPK, (Eds.), Mansfeld’s encyclopedia of agricultural and horticultural crops (vol. 3, pp. 1141–1150). Springer.

[fsn32535-bib-0018] Hammer , Ø. , Harper , D. A. T. , & Ryan , P. D. (2001). PAST: Paleontological statistics software package for education and data analysis. Palaeontologia Electronica, 4(1), 9. http://palaeoelectronica.org/2001_1/past/issue1_01.htm

[fsn32535-bib-0019] Hemati, A. , Azarnia, M. , & Angaji, A. H. (2010). Medicinal effects of *Heracleum persicum* (Golpar). Middle‐East Journal of Scientific Research, 5(3), 174–176.

[fsn32535-bib-0020] Himesh, S. , Sarvesh, S. , Sharan, P. , & Singhai, A. (2011). Preliminary phytochemical screening and HPLC analysis of flavonoid from methanolic extract of leaves of *Annona squamosa* . International Research Journal of Pharmacy., 2(5), 242–246.

[fsn32535-bib-0021] Hosseini, A. S. , Akramian, M. , Khadivi, A. , & Salehi‐Arjmand, H. (2018). Phenotypic and chemical variation of black mulberry (*Morus nigra*) genotypes. Industrial Crops and Products, 117, 260–271.

[fsn32535-bib-0022] Johnston, M. C. (1963). The species of *Ziziphus* indigenous to United States and Mexico. American Journal of Botany, 50, 1020–1027. 10.1002/j.1537-2197.1963.tb06585.x

[fsn32535-bib-0023] Jongbloed, M. (2003). The comprehensive guide to the wild flowers of the United Arab Emirates. Environmental Research and Wildlife Development Agency (ERWDA).

[fsn32535-bib-0024] Karadeniz, T. (2002). Selection of native ‘cornelian’ cherries grown in Turkey. Journal of the American Pomological Society, 56, 164–167.

[fsn32535-bib-0025] Kaurinovic, B. , Popovic, M. , Vlaisavljevic, S. , & Trivic, S. (2011). Antioxidant capacity of *Ocimum basilicum* L. and *Origanum vulgare* L. extracts. Molecules, 16, 7401–7414. 10.3390/molecules16097401 21878860 PMC6264430

[fsn32535-bib-0026] Khadivi, A. (2018). Phenotypic characterization of *Elaeagnus angustifolia* using multivariate analysis. Industrial Crops Products, 120, 155–161.

[fsn32535-bib-0027] Khadivi‐Khub, A. , & Anjam, K. (2014). Morphological characterization of *Prunus scoparia* using multivariate analysis. Plant Systematics and Evolution, 300, 1361–1372.

[fsn32535-bib-0028] Krishna, H. , Singh, D. , Singh, R. S. , Kumar, L. , Sharma, B. D. , & Saroj, P. L. (2020). Morphological and antioxidant characteristics of mulberry (*Morus* spp.) genotypes. Journal of the Saudi Society of Agricultural Sciences, 19, 136–145.

[fsn32535-bib-0029] Lin, S. , Traver, D. , Zhu, H. , Dooley, K. , Paw, B. , & Zon, R. L. (2005). Analysis of thrombocyte development in CD41‐GFP transgenic zebrafish. Blood, 106, 3803–3810. 10.1182/blood-2005-01-0179 16099879 PMC1895094

[fsn32535-bib-0030] Mallavadhani, U. , Sudhakar, A. , Sathyanarayana, K. , Mahapatra, A. , Li, A. , & Richard, B. (2006). Chemical and analytical screening of some edible mushrooms. Food Chemistry, 95, 58–64.

[fsn32535-bib-0048] Markovski, A. , & Velkoska‐Markovska, L. (2015). Investigation of the morphometric characteristics of jujube types (*Ziziphus jujuba* Mill.) fruits in Republic of Macedonia. Genetika, 47, 33–43. 10.2298/gensr1501033m

[fsn32535-bib-0031] Maydell, H. J. (1986). Trees and shrubs of the Sahel: Their characteristics and uses. Deutsche Gesellschaft fur Technische Zusammenarbeit (GTZ) GmbH.

[fsn32535-bib-0032] Miehe, S. (1986). Acacia albida and other multipurpose trees on the fur farmlands in the Jebbel Marra highlands, western Darfur, Sudan. Agroforestry Systems, 4, 89–119.

[fsn32535-bib-0033] Norouzi, E. , Erfani‐Moghadam, J. , Fazeli, A. , & Khadivi, A. (2017). Morphological variability within and among three species of *Ziziphus* genus using multivariate analysis. Scientia Horticulturae, 222, 180–186. 10.1016/j.scienta.2017.05.016

[fsn32535-bib-0034] Norusis, M. J. (1998). SPSS/PC advanced statistics. SPSS Inc.

[fsn32535-bib-0035] Obeid, M. , & Mahmoud, A. (1971). Ecological studies in the vegetation of the Sudan: II. The ecological relationships of the vegetation of Khartoum province. Vegetatio, 23, 77–198. 10.1007/BF02350621

[fsn32535-bib-0036] Sachez‐Salcedo, E. M. , Mena, P. , Garcia‐Viguera, C. , Martinez, J. J. , & Hernandez, F. (2015). Phytochemical evaluation of white (*Morus alba* L.) and black (*Morus nigra* L.) mulberry fruits, a starting point for the assessment of their beneficial properties. Journal of Functional Foods, 12, 399–408.

[fsn32535-bib-0037] Saied, A. S. , Gebauer, J. , Hammer, K. , & Buerkert, A. (2008). *Ziziphus spina‐christi* (L.) Willd.: A multipurpose fruit tree. Genetic Resources and Crop Evolution, 55(7), 929–937.

[fsn32535-bib-0038] SAS Institute . (1990). SAS® Procedures Version 6 (3rd ed.). North Carolina, USA: SAS Institute.

[fsn32535-bib-0039] Schreckenberg, K. , Awono, A. , Degrande, A. , Mbosso, C. , Ndoye, O. , & Tchoundjeu, Z. (2006). Domesticating indigenous fruit trees as a contribution to poverty reduction. Forests Trees Livelihoods, 16, 35–51. 10.1080/14728028.2006.9752544

[fsn32535-bib-0040] Schubert, A. , Pereira, D. , Zanin, F. , Alves, S. , Beck, R. , & Athayde, M. (2007). Comparison of antioxidant activities and total polyphenolic and methylxanthine contents between the unripe fruit and leaves of Ilex paraguariensis A. St. Hil. Die Pharmazie‐An International Journal of Pharmaceutical Sciences, 62, 876–880. 18065107

[fsn32535-bib-0041] Singleton, V. L. , & Rossi, J. A. (1965). Colorimetry of total phenolics with phosphomolybdic‐phosphotungstic acid reagents. American Journal of Enology and Viticulture, 16, 144–158.

[fsn32535-bib-0042] Tanaka, M. , Kuie, C. , Nagashima, Y. , & Taguchi, T. (1998). Applications of antioxidative maillard reaction products from histidine and glucose to sardine products. Nippon Suisan Gakaishi, 54, 1409–1414.

[fsn32535-bib-0043] Turkmen, N. , Sari, F. , & Velioglu, Y. (2006). Effects of extraction solvents on concentration and antioxidant activity of black and black mate tea polyphenols determined by ferrous tartrate and Folin‐Ciocalteu methods. Food Chemistry, 99, 835–841.

[fsn32535-bib-0044] Vogt, K. (1995). A field worker’s guide to the identification, propagation and uses of common trees and shrubs of dryland Sudan. SOS Sahel International.

[fsn32535-bib-0045] Weekley, C. W. , Kubisiak, T. L. , & Race, T. M. (2002). Genetic impoverishment and cross‐incompatibility in remnant genotypes of *Ziziphus celata* (Rhamnaceae), a rare shrub endemic to the Lake Wales Ridge, Florida. Biodiversity & Conservation, 11, 2027–2046.

[fsn32535-bib-0046] Zheng, W. Z. , & Wang, S. Y. (2001). Antioxidant activity and phenolic compounds in selected herbs. Journal of Agricultural and Food Chemistry, 49(11), 5165–5170. 10.1021/jf010697n 11714298

[fsn32535-bib-0047] Zhu, Z. , Liang, Z. , & Han, R. (2009). Saikosaponin accumulation and antioxidative protection in drought‐stressed *Bupleurum chinense* DC. Plants. Environmental and Experimental Botany, 66, 326–333.

